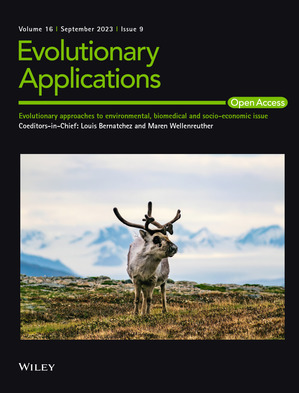# Cover Image

**DOI:** 10.1111/eva.13409

**Published:** 2023-09-25

## Abstract

Caption: A Svalbard reindeer grazing on the summer tundra in Adventdalen, Svalbard. In the background are the mountains and glaciers on the north side of Isfjorden, where reindeer were reintroduced to from Adventdalen.

Credit: Hamish Burnett.